# Predictions of heading date in bread wheat (*Triticum aestivum* L.) using QTL-based parameters of an ecophysiological model

**DOI:** 10.1093/jxb/eru328

**Published:** 2014-08-22

**Authors:** Matthieu Bogard, Catherine Ravel, Etienne Paux, Jacques Bordes, François Balfourier, Scott C. Chapman, Jacques Le Gouis, Vincent Allard

**Affiliations:** ^1^INRA, UMR 1095 Génétique, Diversité et Ecophysiologie des Céréales, 5 chemin de Beaulieu, F-63039 Clermont-Ferrand, France; ^2^Université Blaise Pascal, UMR 1095 Génétique, Diversité et Ecophysiologie des Céréales, F-63177 Aubière Cedex, France; ^3^CSIRO, Queensland Bioscience Precinct - St Lucia, 306 Carmody Road, St Lucia QLD 4067, Australia

**Keywords:** Association genetics, ecophysiological model, gene-based modelling, heading date, phenology, wheat.

## Abstract

Prediction of wheat phenology facilitates the selection of cultivars with specific adaptations to a particular environment. However, while QTL analysis for heading date can identify major genes controlling phenology, the results are limited to the environments and genotypes tested. Moreover, while ecophysiological models allow accurate predictions in new environments, they may require substantial phenotypic data to parameterize each genotype. Also, the model parameters are rarely related to all underlying genes, and all the possible allelic combinations that could be obtained by breeding cannot be tested with models. In this study, a QTL-based model is proposed to predict heading date in bread wheat (*Triticum aestivum* L.). Two parameters of an ecophysiological model (*V*_*sat*_ and *P*_*base*_, representing genotype vernalization requirements and photoperiod sensitivity, respectively) were optimized for 210 genotypes grown in 10 contrasting location × sowing date combinations. Multiple linear regression models predicting *V*_*sat*_ and *P*_*base*_ with 11 and 12 associated genetic markers accounted for 71 and 68% of the variance of these parameters, respectively. QTL-based *V*_*sat*_ and *P*_*base*_ estimates were able to predict heading date of an independent validation data set (88 genotypes in six location × sowing date combinations) with a root mean square error of prediction of 5 to 8.6 days, explaining 48 to 63% of the variation for heading date. The QTL-based model proposed in this study may be used for agronomic purposes and to assist breeders in suggesting locally adapted ideotypes for wheat phenology.

## Highlight text

QTL-based parameters of an ecophysiological model, calibrated on an association genetics panel of 210 genotypes, allowed prediction of heading dates of 80 independent genotypes in six independent experiments with a median prediction error of 5.6 days.

## Introduction

Hexaploid wheat is native to the Middle East and now occupies 22% of the total cultivated area in the world (Leff *et al.*, 2004). Much of this expansion in cultivation area has been facilitated by developing adapted wheat cultivars through the use of genetic variation for the timing of plant development or phenology (Cockram *et al.*, 2007). Short-cycle spring wheat cultivars have been selected for regions with a highly continental climate that require late planting in the spring to avoid frost damage and early harvesting to avoid drought or heat stress in summer. In more temperate climatic regions, long-cycle cultivars allow planting in autumn to benefit from a longer growing season. These adaptations have resulted from both historical selection of seed by farmers over centuries of local production, and over the last century or so, from breeder selections for phenological variation, particularly for heading date

By influencing the time that a crop has to use resources, phenology accounts for substantial genetic control of grain yield in cultivated plant species. In wheat, the fine tuning of cultivar earliness is a prerequisite for matching climatic conditions and realising yield potential in a given environment (Snape *et al.*, 2001; Reynolds *et al.*, 2009; Foulkes *et al.*, 2011). In the context of changes in global climate that are associated with an increase in the occurrence and intensity of drought and heat stress in the main areas of wheat cultivation (Lobell *et al.*, 2011; Semenov and Shewry, 2011), strategies based on adaptation through earliness are often proposed to avoid these constraints (Gouache *et al.*, 2012; Zheng *et al.*, 2012). In cultivated conditions, wheat susceptibility to biotic and abiotic stress (Slafer and Rawson, 1995; Porter and Semenov, 2005; Del Ponte *et al.*, 2007) and fertilizer requirements (Hirel *et al.*, 2007; Pask *et al.*, 2012) varies during the life cycle. Reciprocally, the efficiency of cultural management varies during the crop life cycle. For example, heading date is usually taken as a reference point for the last nitrogen fertilization application as this increases grain protein concentration without decreasing grain yield (Gooding and Davies, 1992; Woolfolk *et al.*, 2002). Therefore, predicting phenology of wheat cultivars is essential for choosing the best-adapted cultivar for a specific growing region and to plan cultural management at the right time.

Wheat phenology depends on vernalization requirements, photoperiod sensitivity, and earliness *per se* (Worland, 1996). Substantial progress has been made in understanding the genetic control of wheat phenology, and several major genes have been cloned. The *Ppd-1* genes, located on the homoeologous group 2, are involved in the circadian clock (Beales *et al.*, 2007). Mutations at these loci cause semi-dominant photoperiod insensitivity which results in early flowering. Photoperiod-insensitive alleles have been identified for each of the three homoeologous copies (Mohler *et al.*, 2004; Beales *et al.*, 2007; Wilhelm *et al.*, 2009), conferring different degrees of photoperiod insensitivity. The allele denoted *Ppd-D1a* creates the earliest-flowering phenotype, followed by *Ppd-A1a* and *Ppd-B1a* (Bentley *et al.*, 2010). The major effects of vernalization requirements are determined by the *Vrn* genes (Worland, 1996; Snape *et al.*, 2001). The *Vrn-1* series (on homoeologous group 5) are involved in the perception of the vernalization signal and the induction of the floral transition at the shoot apex (Yan *et al.*, 2003). Mutations (insertions or deletions) in the regulatory regions of at least one of the three homoeologous *Vrn-1* copies are associated with dominant spring growth alleles (Fu *et al.*, 2005). The *Vrn-2* series are located on homoeologous group 4 (*Vrn-B2* and *Vrn-D2*) and 5 (*Vrn-A2*) (Yan *et al.*, 2004), and act as flowering repressors which are downregulated by vernalization (Yan *et al.*, 2004). The *Vrn-3* series, located on homoeologous group 7, has been identified as the ‘florigen’ signal moving from leaves to promote the floral transition at the shoot apex (Yan *et al.*, 2006; Li and Dubcovsky, 2008). Mutations (SNP, insertions-deletions) on the *Vrn-D3* series have been shown to induce variations for heading date (Bonnin *et al.*, 2008). A *Vrn-D4* gene, located in the centromeric region of chromosome 5D, has also been identified (Yoshida *et al.*, 2010). Molecular studies have unravelled interactions among these major genes, and gene networks showing their inter-relationship have been proposed (Trevaskis *et al.*, 2007; Distelfeld *et al.*, 2009; Shimada *et al.*, 2009; Trevaskis, 2010). No major functional genes have yet been identified with earliness *per se*, the characteristic whereby an ‘early’ line will flower more quickly than a ‘late’ line if both are first vernalized and then grown under an extended photoperiod regime.

Numerous QTL studies for heading date have been carried out in wheat (e.g. Sourdille *et al.*, 2000; Börner *et al.*, 2002; Hanocq *et al.*, 2004; Bogard *et al.*, 2011; Carter *et al.*, 2011). This growth stage is relatively easier to measure compared to other critical growth stages such as the beginning of stem elongation or anthesis. Although heading date correlates with anthesis date, genetic variability for the heading to anthesis date period exists in wheat (unpublished results) and would probably require specific studies. Apart from the major genes described above, the combined analysis of these QTL studies or meta-QTL analysis have revealed small-effect loci on most of the wheat chromosomes (Hanocq *et al.*, 2007; Griffiths *et al.*, 2009). However, while these studies identify novel loci, their utility is limited in terms of prediction. Indeed, QTL analysis of heading date in multiple environments leads to an ‘environment-specific’ QTL model as significant QTLs and estimated additive effects differ with the environments. For example, in a study of a wheat population grown in 10 environments, the estimated additive effect of the *Ppd-D1* gene varied from 5 to 9 days depending on the environment (Bogard *et al.* 2011).

Ecophysiological models account for both environment and genetic effects through a design process that aims to dissect traits into ‘environment-independent’ components. Genotype × environment interactions then become emergent properties of the models (Chapman *et al.*, 2003; Trewavas, 2006; Bertin *et al.*, 2010; Yin and Struik, 2010). The use of ecophysiological models allows prediction of wheat with a good accuracy with root mean square error of prediction (RMSEP) for heading date being as low as 4 to 7 days (Weir *et al.*, 1984; Asseng *et al.*, 1998; White *et al.*, 2008; He *et al.*, 2011; Zheng *et al.*, 2013). Ecophysiological models that predict wheat phenology include: (1) Empirical models based on accumulation of thermal time modified by vernalization and photoperiod (Weir *et al.*, 1984; Ritchie and Otter, 1985; Asseng *et al.*, 1998) and (2) mechanistic models simulating the emission of leaves and spike primordia at the shoot apex (Jamieson *et al.*, 1998). Empirical models rely on the daily calculation of vernalization and photoperiod factors reducing daily thermal time accumulation; once the total accumulated thermal time passes a defined value, a given stage is reached. In the mechanistic model Sirius (Jamieson *et al.*, 1998), photoperiod affects the final leaf number in response to the daylength occurring at the two-leaf stage after the flag-leaf primordium has formed [see details in Brooking *et al.* (1995)]. The vernalization submodel in Sirius was described in Robertson *et al.* (1996). Briefly, a vernalization index is calculated daily depending on daily mean temperature and cardinal vernalizing temperatures (vernalizing effects increasing linearly from 0 to 15°C and decreasing linearly to 0 from 15 to 17°C). Vernalization is satisfied either when the vernalization index reaches one or when the number of primordia on the apex exceeds a maximal attainable final leaf number corresponding to the number of leaves produced by a winter cultivar grown in long days at temperatures above 17°C. Although these models differ in form, their performances in terms of prediction are comparable (Jamieson *et al.*, 2007). In addition to agro-climatic inputs (e.g. temperature, photoperiod, sowing date, and soil type), response parameters are required to initialize these models. Some of these, termed ‘genetic parameters’, reflect genetic variations in the model and may differ among cultivars. An important property of these genetic parameters is their assumed *de facto* independence from the environment. Unfortunately, determining these parameters’ values requires extensive and time-consuming experiments for each cultivar either for them to be measured (when this is feasible) or to be optimized.

The concept of a gene-based model has been proposed to describe the possible future direction of crop modelling to capture the large amount of data generated by molecular techniques (White and Hoogenboom, 1996; Chapman *et al.*, 2002a; Chapman *et al.*, 2002b; White and Hoogenboom, 2003; White, 2006; Chapman, 2008; Letort *et al.*, 2008; White, 2009). Gene-based modelling aims to fill the gap between ecophysiological modelling and genetics by integrating knowledge from each field into a holistic framework. The approach consists of replacing empirically determined genetic parameters by genetic coefficients explicitly reflecting the allelic composition of the genotypes. For crop improvement, one output of gene-based ecophysiological models would be the *in silico* identification of the best allelic combination for a given set of environments, so-called ‘ideotypes’ (Reymond *et al.*, 2003; Letort *et al.*, 2008; Chenu *et al.*, 2009). Examples of gene-based or QTL-based models include: leaf elongation rate in *Zea mays* (Reymond *et al.*, 2003; Chenu *et al.*, 2008); *Prunus* quality (Quilot *et al.*, 2005); phenology in *Glycine max* (Messina *et al.*, 2006); flowering date in *Oryza sativa* (Nakagawa *et al.*, 2005); barley (Yin *et al.*, 2005); *Phaseolus vulgaris* (White and Hoogenboom, 2003); wheat (White *et al.*, 2008); *Brassica oleracea* (Uptmoor *et al.*, 2011); and using knowledge on the gene network regulating flowering in wheat (Brown *et al.*, 2013). Particularly relevant to the research domain here, White *et al.* (2008) proposed a gene-based model to predict the genetic parameters of the CSM-CROPSIM-CERES model for wheat cultivars using multiple linear regressions with genetic markers for *Ppd-D1* and *Vrn1* genes as predictors. They concluded that, if gene-based prediction of wheat phenology appeared feasible, more genetic information should be included into the model. More recently, Zheng *et al.* (2013) estimated the effect of *Ppd-D1* and *Vrn1* genes on two phenological parameters of the APSIM model (Keating *et al.*, 2003) and obtained high predictive ability for spring wheats grown across the Australian wheat belt. However, their method still requires that experiments be carried out under artificial conditions (artificial vernalization and extended photoperiod) to estimate a third genotype-specific parameter related to the duration in modified thermal time between emergence and heading, and is therefore not a method that can be applied using only genetic information.

The objective of this study is to propose a QTL-based ecophysiological model to predict heading date in bread wheat (*Triticum aestivum* L.). In contrast with the approaches of White *et al.* (2008) and Zheng *et al.* (2013), no assumptions were made about which genes determined the model parameters. The strategy consisted in optimizing two genetic parameters of an ecophysiological model (*V*_*sat*_ and *P*_*base*_, representing vernalization requirement and photoperiod sensitivity, respectively) for each of the 210 genotypes of an association genetics panel grown under contrasting conditions. Genetic markers associated with model parameters were then identified and used to obtain multiple linear models predicting *V*_*sat*_ and *P*_*base*_ by stepwise regression. Finally, predictions of heading dates using QTL-based parameters were tested on an independent set of 88 genotypes grown in six environments.

## Materials and methods

### Phenotypic data

Heading dates were recorded in various contrasting conditions for a large panel of bread wheats comprising different combinations of spring/winter and photoperiod sensitive/insensitive accessions. Association genetics analyses have been published using this panel to study earliness components (Bonnin *et al.*, 2008; Rousset *et al.*, 2011; Le Gouis *et al.*, 2012). The panel used here is a subset of 210 accessions from the INRA bread wheat core collection of 372 accessions which aims to represent worldwide wheat diversity (Balfourier *et al.*, 2007). The subset aimed to maintain the diversity originally present in the entire panel (Rousset *et al.*, 2011). Experiments were carried out in France in different location × sowing date combinations. The association genetics panel was grown for three autumn sowings and one spring sowing at Clermont-Ferrand (45°46’N, 03°09’E, 401m a.s.l.) and three autumn sowings and three spring sowings at Le Moulon (48°42’ N, 02°08’ E, 156m a.s.l.) as described by Bonnin *et al.* (2008), Rousset *et al.* (2011) and Le Gouis *et al.* (2012). Autumn-sown experiments in Le Moulon (2003 to 2005) were sown in two randomized complete blocks where 20 seeds of each genotype were sown in two 1.2-m-long single rows. In the other experiments, 10 seeds of each genotype were sown in a single-row. Ear emergence day of the main tiller of five to six individual plants was recorded when half of the ear had emerged from the flag leaf. Numbers were averaged to obtain heading date for each genotype, reported here as day of the year (DOY).

### Ecophysiological model

The ecophysiological model used in this study is based on the accumulation of thermal time modified by vernalization and photoperiod factors. The model works on a daily time-step and calculates, for each day, the thermal time accumulated as a function of daily temperature and daily photoperiod. Wheat development is split into different phases, and three factors limited to vary between 0 and 1 reducing thermal time accumulation are calculated based on response curves for temperature, accumulated vernalizing days, and photoperiod (FT, FV, and FP, respectively). Daily accumulated thermal time (Tt) is modified by vernalization and photoperiod factors (FV and FP) during the phase from emergence to heading and leads to the calculation of a modified thermal time (PVTt):

$$\begin{array}{l} \text{PVTt}=\text{Tt}\times \text{FV}\times \text{FP} \end{array}$$ (1)

A growth stage is reached when the accumulated modified thermal time exceeds the duration of the corresponding phase. The duration of the sowing to emergence phase was 148 modified degree days, and the duration from emergence to heading (*TT*_*emhe*_) was either fixed at 500 modified degree days or allowed to vary between genotypes depending on the optimization strategy (see ‘Optimization of model parameters’ section).

The model is a modified version of that proposed by Weir *et al.* (1984). The original form relied on the simulation of a cosinusoidal variation of temperature across the day based on daily minimal and maximal temperature data. The day is divided into eight three-hours periods, and the contribution of temperature across the day is then integrated by averaging the contributions of the eight periods [equation 1 in Weir *et al.* (1984)]. In contrast, the present model used daily mean temperature (*TM*) only:

$$\;TM=\frac{\left(Tmin+Tmax\right)}{2}$$ (2)

with Tmin being the daily minimum temperature, and Tmax the daily maximum temperature. *FT* is calculated from *TM* and a bilinear function with three cardinal temperatures (*T*_*base*_, *T*_*opt*_, and *T*_*max*_):

$$FT=0\;for\;TM\leq T_{base\;}\text{ or }TM\geq T_{max}$$ (3)

$$FT=\frac{\left(TM-T_{base}\right)}{\left(T_{opt}-T_{base}\right)}\;for\;T_{base}<TM\leq T_{opt}$$ (4)

$$FT=\frac{\left(T_{max}-TM\right)}{\left(T_{max}-\;T_{opt}\right)}\text{ for }T_{opt}<TM\leq T_{max}$$ (5)

*T*_*base*_, *T*_*opt*_, and *T*_*max*_ were set to 1°C, 26°C, and 37°C, respectively, as in Weir *et al.* (1984). *FT* allows calculation of the contribution of *TM* to the daily accumulated thermal time (*Tt*):

$$Tt=FT\times T_{opt}$$ (6)

The vernalization factor *FV* is calculated depending on the number of vernalizing days (*VDD*) accumulated by the crop. *VDD* accumulates from germination, although *FV* modifies the daily accumulated thermal time only from plant emergence with the effect continuing until heading is reached. This is a simplification of the model proposed by Weir *et al.* (1984) in which the vernalization effect stops at floral initiation. Each day, the efficiency of vernalization (*V*_*eff*_) is calculated from *TM* and a function with four cardinal temperatures (*T*_*1*_, *T*_*2*_, *T*_*3*_, and *T*_*4*_):

$$V_{eff}=0\text{ for }TM<T_{1}\text{ or }TM>T_{4}$$ (7)

$$V_{eff}=\frac{\left(TM-T_{1}\right)}{\left(T_{2}-T_{1}\right)}\text{for }T_{1}\leq TM<T_{2}$$ (8)

$$V_{eff}=1\;\text{for}T_{2}\leq TM\leq T_{3}\;\;\text{for }T_{2}\leq TM\leq T_{3}$$ (9)

$$V_{eff}=\frac{\left(T_{4}-TM\right)}{\left(T_{4}-T_{3}\right)}\text{for }T_{3}<TM\leq T_{4}$$ (10)

*T*_*1*_, *T*_*2*_, *T*_*3*_, and *T*_*4*_ were set to –4°C, 3°C, 10°C, and 17°C, respectively, as in Weir *et al.* (1984). *VDD* is then calculated by summing *V*_*eff*_ from day 1 to day i:

$$VDD_{i}=\overset{i}{\underset{1}{\sum }}V_{eff}$$ (11)

*FV* varies between 0 and 1 and is calculated from *VDD* and a function with two parameters, *V*_*base*_ and *V*_*sat*_:

$$FV=\frac{\left(VDD-V_{base}\right)}{\left(V_{sat}-V_{base}\right)}$$ (12)

*V*_*base*_ was set to zero days so that spring wheats with *V*_*sat*_ close to zero reach full vernalization (FV = 1) very early. *V*_*sat*_ was considered as a genetic parameter, thus varying between genotypes.

Daily photoperiod was calculated as in Weir *et al.* (1984) by considering that photoperiod-effective radiation starts and ends when the sun is 6° below the horizon. The photoperiod factor (*FP*) is calculated from daily photoperiod (*Ph*) and a function with two parameters, *P*_*opt*_ and *P*_*base*_. *FP* is limited to vary between 0 and 1 and is calculated as:

$$FP=\frac{\left(Ph-P_{base}\right)}{\left(P_{opt}-P_{base}\right)}$$ (13)

*P*_*opt*_ was set to 20h while *P*_*base*_ was considered as a genetic parameter. The model was coded in S language and run using R (R Development Core Team, 2013).

### Optimization of model parameters

Either two (*V*_*sat*_ and *P*_*base*_, *2p* strategy) or three (*V*_*sat*_, *P*_*base*_, and *TT*_*emhe*_ *, 3p* strategy) parameters representing different earliness components were optimized. Regarding photoperiod sensitivity, *P*_*base*_ or *P*_*opt*_ could have been optimized indifferently (equation 13). The choice of *P*_*base*_ was therefore arbitrary. Regarding vernalization requirement, cardinal vernalizing temperatures could have been optimized instead of *V*_*sat*_, but this would probably have required optimizing at least two temperatures (T1 and T2 in equation 7 to 9) and therefore increased the number of parameters to optimize. In the same way, it appeared more relevant to optimize *TT*_*emhe*_, representing earliness *per se*, instead of cardinal temperatures *T*_*base*_, *T*_*opt*_, or *T*_*max*_.

*P*_*base*_ was varied between 0 and 10h with a 0.1h step (101 values); *V*_*sat*_, between 0 and 130 days with a 1 day step (131 values); and *TT*_*emhe*_, between 400 and 800 with a 10 modified degree days step (41 values). In the *2p* strategy, *TT*_*emhe*_ was fixed at 500 modified degree days. All the *V*_*sat*_ × *P*_*base*_ (*2p* strategy) or *V*_*sat*_ × *P*_*base*_ × *TT*_*emhe*_ (*3p* strategy) combinations were generated leading to 101×131 = 13 231 or 101×131×41 = 542 471 parameter combinations (or ‘parameters vectors), for *2p* and *3p*, respectively. Results obtained with the *2p* and *3p* strategies were compared. Additionally, *V*_*sat*_ and *P*_*base*_ were optimized for the original and the modified version of the Weir *et al.* (1984) model, and results of predictions obtained for the calibration data set were compared to check if our modified version for temperature did not reduce model predictive ability.

For each genotype, parameter optimization was carried out by simulating predicted heading dates for all the *V*_*sat*_ × *P*_*base*_ (*2p* strategy) or *V*_*sat*_ × *P*_*base*_ × *TT*_*emhe*_ (*3p* strategy) combinations, for all experiments of the calibration data set where the genotype was tested, and then calculating the RMSEP between observed and predicted heading dates:

$$RMSEP_{i}=\sqrt{\frac{\sum_{i=1}^{n}{\left(Obs_{ij}-Pred_{ij}\right)}^{2}}{n}}$$ (14)

where $Obs_{ij}$ and $Pred_{ij}$ are the observed and predicted heading dates of genotype *i* in DOY for experiment *j*, and *n* is the number of experiments where genotype *i* was tested. For each genotype, the vector(s) of parameters that resulted in the minimum RMSEP across environments was (were) selected.

In order to link parameters to genetic markers, it is necessary to unambiguously identify parameter vectors that not only allow accurate prediction of heading date but also reflect the genetic architecture of the accessions making up the panel used in the study. The panel used in this study may be split into 53 winter and 157 spring genotypes, the former being genotypes that did not head in the spring-sown experiments and apparently had an obligate requirement for vernalisation. Parameter optimization was conducted differently for these two groups. Optimizations of spring genotypes were carried out using autumn- and spring-sown experiments, while optimizations of winter genotypes were carried out using autumn-sown experiments only. As results of optimization were ambiguous in this latter case, with multiple parameter vectors minimizing the RMSEP, the information that winter genotypes did not head under spring sowing was used to filter parameter vectors. This filtering consisted in removing sets of parameters that predicted heading dates which were earlier than the last heading date recorded plus 10 days in each of the spring-sown experiments. When multiple parameter vectors led to the minimum RMSEP, a vector of parameters was arbitrarily chosen so that *P*_*base*_ and *V*_*sat*_ were close to the median values of the different parameter vectors minimizing the RMSEP.

An additional analysis was performed to test the robustness of *P*_*base*_ and *V*_*sat*_ optimization (*2p* strategy). The procedure consisted in optimizing *P*_*base*_ and *V*_*sat*_ for each genotype using different sets of experiments. Each set of experiments was obtained for each genotype by sampling at random n_spring–1_ of the spring-sown experiments and n_autumn–1_ of the autumn-sown experiments (n_spring_ and n_autumn_ being the total number of available spring or autumn experiments for a given genotype). For each genotype, optimizations were carried out separately for each random set of experiments, and results were compared to assess the robustness of the *V*_*sat*_ and *P*_*base*_ parameters.

### Model sensitivity analysis

A basic sensitivity analysis was carried out by running simulations of heading date for all the 542 471 *P*_*base*_ × *V*_*sat*_ × *TT*_*emhe*_ parameter combinations for each autumn- and spring-sown experiment of the calibration data set. Variations of heading date for each experiment were modelled as a multiple linear model with *V*_*sat*_, *P*_*base*_, and *TT*_*emhe*_ as predictors:

$$\text{Y}=\beta_{0}+\overset{\text{p}}{\underset{\text{i=1}}{\sum }}\beta_{\text{i}}{\text{X}}_{\text{i}}$$

The variation of heading date due to each parameter was assessed using standardized regression coefficients (SRC) calculated as follows:

$${\text{SRC}}_{\text{i}}=\frac{\beta^{2}\text{V}\left({\text{X}}_{\text{i}}\right)}{\text{V}\left(\text{Y}\right)}$$

with

$$\text{V}\left(\text{Y}\right)=\overset{\text{p}}{\underset{\text{i}=1}{\sum }}\beta^{2}\text{V}\left({\text{X}}_{\text{i}}\right)$$

where Y represents simulated heading dates; X_i,_ values of parameter i (*P*_*base*_, *V*_*sat*_, or *TT*_*emhe*_); β_0,_ the intercept; and β_i,_ regression coefficient for parameter i. This showed (as a percentage) how much variation of heading date was due to *V*_*sat*_, *P*_*base*_, or *TT*_*emhe*_. The SRC were then aggregated for the autumn-and spring-sown experiments to show differences in model sensitivity.

### Genotype data

Genotype imputation was carried out for 1797 (SSR, DArT, and SNP) unmapped markers by random forest regression for all the genotypes (Stekhoven and Bühlmann, 2012). Genotype imputation allows missing genotype data to be filled by using genetic information from relatives. Aside from methods requiring physical or genetic map information, random forest regression allows imputation of genotype data without prior knowledge of marker positions. Analysis of imputed data should lead to increased statistical power for association genetics, but in this study is essential to allow fitting of statistical models linking parameters to genetic markers on the whole set of genotypes (otherwise, genotypes with missing data for any of the markers involved in the model would have been discarded). Genotype imputation of the unordered markers was carried out in R using the missForest package (Stekhoven and Bühlmann, 2012). The missForest function provides the proportion of falsely classified (PFC) entries for each marker. Markers for which genotype imputation resulted in a PFC > 0.2 were discarded before subsequent regression analysis. This resulted in 1603 polymorphic genetic markers including 53 SSR, 451 DArT, and 1099 SNP.

### Association genetic analysis

Association analysis was carried out in order to identify genetic markers associated to variation of *V*_*sat*_, *P*_*base*_, and *TT*_*emhe*_ for the *2p* and *3p* strategies. For each marker, rare genotypes (frequency less than 5%) were considered as missing data. This led to a missing data rate ranging from 0 to 9% with only 5% of the markers showing some missing data. The structure was obtained by Rousset *et al.* (2011) with 82 SSR markers and was further used by Le Gouis *et al.* (2012). The structure of the panel was taken into account by using the relative contribution of each genotype to four ancestral groups as covariates in the model. Association genetics was carried out for each marker by fitting a linear model as follow:

$$y_{ij}=m_{ik}+g1_{i}+g2_{i}+g3_{i}+g4_{i}+\epsilon_{ij}$$ (15)

where y_ij_ is the value of trait j (*V*_*sat*_ *, P*_*base*_, or *TT*_*emhe*_) for genotype i, m_ik_ is the allele of genotype i at marker k, and $g1_{i}+g2_{i}+g3_{i}+g4_{i}$ are the contributions of genotype i to each of four ancestral groups. Association analysis was carried out using TASSEL v2.1 (Bradbury *et al.*, 2007). Adjusted *P*-values were obtained after 1000 permutations.

### Statistical models to predict *V*_*sat*_, *P*_*base*_, and *TT*_*emhe*_

Only genetic markers associated (*P* < 0.05) with one of the model parameters (*V*_*sat*_, *P*_*base*_, and *TT*_*emhe*_) were considered. First, blocks of collinear markers were identified by calculating the correlation coefficient (r_LD_) between all pairs of loci. Markers were considered collinear when r_LD_ ≥ 0.8. Among markers included in a collinear block, the marker with the fewest missing data before genotype imputation was chosen in order to minimize possible errors coming from genotype imputation. Linear models for *V*_*sat*_, *P*_*base*_, and *TT*_*emhe*_ were obtained using multiple linear regressions with backward elimination of the markers: all the markers associated with *V*_*sat*_, *P*_*base*_, or *TT*_*emhe*_ and previously filtered for collinearity were included in the model and markers with *P*-values > 0.05 were iteratively removed one at a time. The same approach was used to model *V*_*sat*_ and *P*_*base*_ using only associated major genes. This analysis quantified the proportion of variation of model parameters that could be explained by additional minor-effect QTLs, compared to known major gene effects.

### Validation of the QTL-based model

The QTL-based model was validated on a set of 88 independent genotypes grown for two years (2006 and 2007) in Estrées-Mons (49°53’N, 3°00’E, 85m a.s.l.), Le Moulon (48°40’N, 2°10’E, 156m a.s.l.), and Joze (45°86’N, 3°30’E, 300m a.s.l.) as described in Bordes *et al.* (2013). These six location × sowing date combinations were not used for model calibration. For each genotype, predicted *V*_*sat*_ and *P*_*base*_ (*2p* strategy) or *V*_*sat*_, *P*_*base*_, and *TT*_*emhe*_ (*3p* strategy) were obtained from the corresponding models linking genetic markers to ecophysiological parameters. The ecophysiological model was then used to predict heading dates for each genotype using parameters as estimated by the genetic markers. Quality criteria used to assess the QTL-based model included the RMSEP between observed and predicted heading dates and the coefficient of determination (R^2^).

## Results

### Model sensitivity analysis

**Fig. 1.**
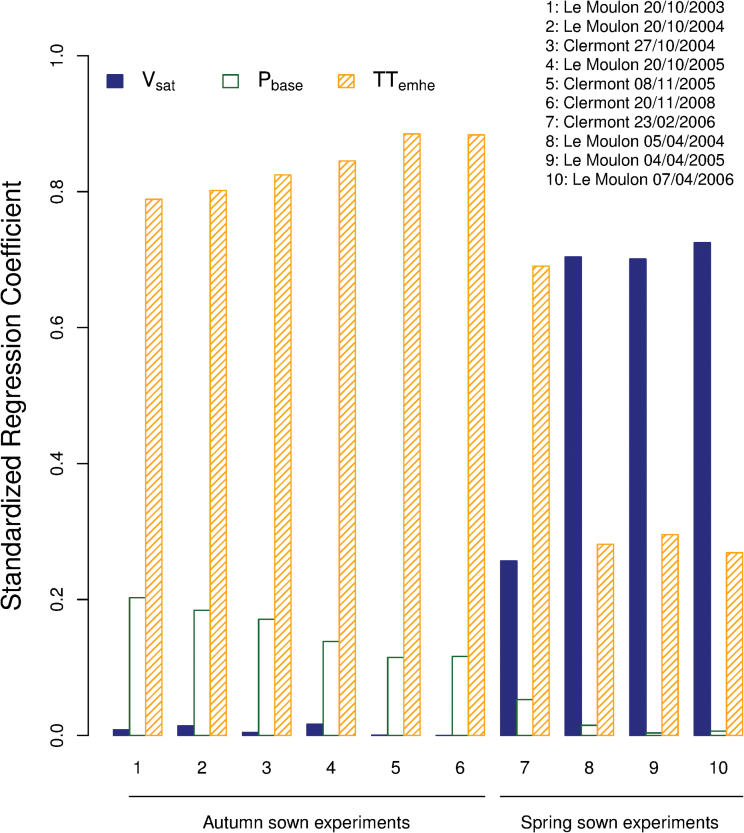
Sensitivity analysis of a modified version of the Weir *et al.* (1984) phenological model. Standardized regression coefficients showing the percentage of variation of heading date explained by each model parameter (*V*_*sat*_, *P*_*base*_, and *TT*_*emhe*_) were calculated in each autumn- and spring-sown experiment used to optimize the model for the genotypes of the calibration data set. This figure is available in colour at *JXB* online.

Standardized regression coefficients (SRC) ranged from 0 to 72%, 0 to 20%, and 27 to 88% for the *V*_*sat*_, *P*_*base*_,and *TT*_*emhe*_ parameters, respectively (Fig. 1). In autumn-sown experiments, the model was much more sensitive to *TT*_*emhe*_ and *P*_*base*_ (SRC ranging from 79 to 88% and 11 to 20%, respectively) than to *V*_*sat*_ (SRC ranging from 0 to 0.01%) (Fig. 1). In contrast, in spring-sown experiments, the model was highly sensitive to *V*_*sat*_ (SRC ranging from 70 to 72%) and less so to *TT*_*emhe*_ (SRC ranging from 27 to 29%) or *P*_*base*_ (SRC ranging from 0 to 0.01%; Fig. 1). These trends were expected as cold temperatures experienced by the crop in autumn-sown experiments generally allows full vernalization, while development of wheat sown under long days in the spring is not expected to be limited by photoperiod.

### Optimization of the ecophysiological model parameters

For each of the 53 winter genotypes (lines that did not head in srping-sown experiments and were parameterized using only autumn-sown experiments), the number of parameter vectors leading to the minimum RMSEP with the *2p* strategy ranged from 1 to 86 and half of the genotypes showed more than eight unique parameter vectors that minimized the RMSEP. Depending on the genotype, standard deviations ranged from 0 to 42 days and 0 to 0.6h for *V*_*sat*_ and *P*_*base*_, respectively. After filtering of these parameter vectors, the number of vectors ranged from 1 to 39 with half of these genotypes having <5 parameter vectors selected. Standard deviations for *V*_*sat*_ and *P*_*base*_ decreased, ranging from 0 to 8 days and 0 to 0.2h for *V*_*sat*_ and *P*_*base*_, respectively. Regarding the 157 spring genotypes for which optimization was carried out using autumn- and spring-sown experiments, the number of parameter vectors selected for each genotype ranged from 1 to 16 and 75% of genotypes had only one unambiguous parameter vector. Standard deviations ranged from 0 to 2.9 days and from 0 to 0.28h for *V*_*sat*_ and *P*_*base*_, respectively. When multiple parameters vectors were found minimizing RMSEP, a vector of parameters was arbitrarily chosen so that *P*_*base*_ and *V*_*sat*_ were close to the median values of the parameter vectors minimizing the RMSEP. As shown by standard deviations of *V*_*sat*_ and *P*_*base*_, for the spring genotypes and after filtering for the winter genotypes, all the possible vectors had relatively similar values for *V*_*sat*_ and *P*_*base*_. Similar results were obtained with the *3p* strategy. Similarly close values of *V*_*sat*_ and *P*_*base*_ were obtained for each genotype when multiple optimizations were carried out using different random sets of experiments (ESM; Fig. 1). Across genotypes, standard deviations of *V*_*sat*_ and *P*_*base*_ ranged from 0 to 12 days and from 0 to 0.4h, respectively. This demonstrated that the optimization procedure was sufficiently robust to reliably parameterize each genotype.

**Fig. 2.**
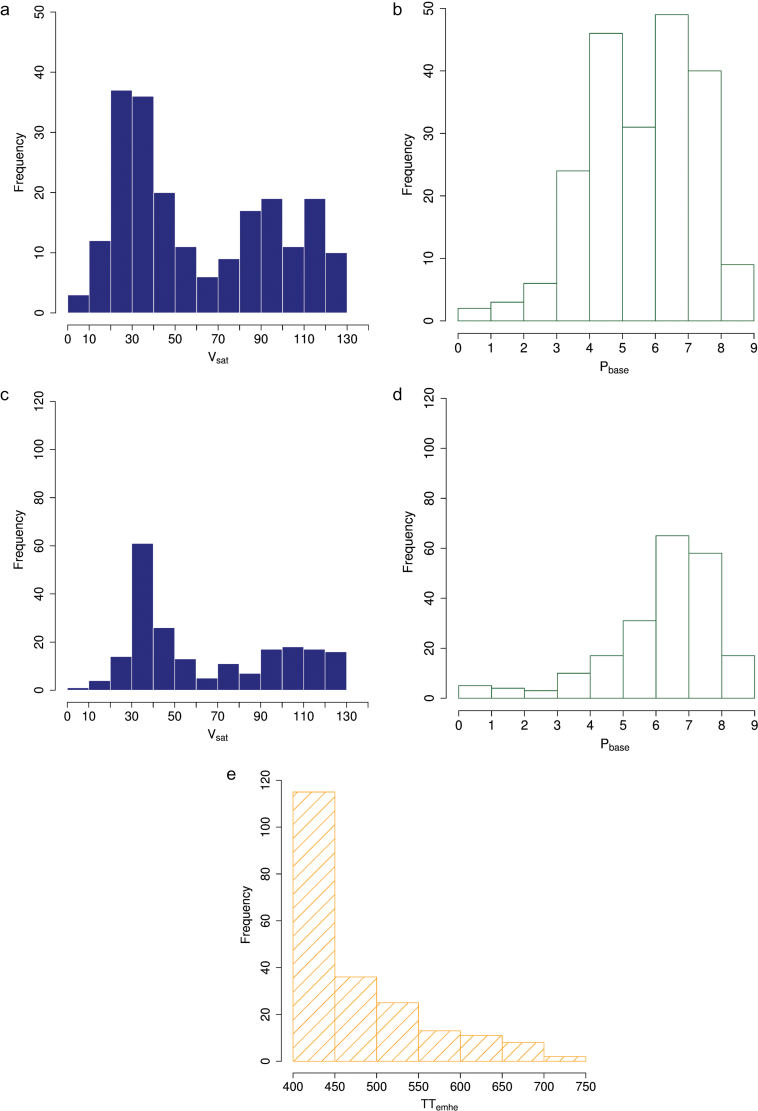
Distributions of the *V*_*sat*_ (a, c), *P*_*base*_ (b, d) and *TT*_*emhe*_ (e) parameters of a modified version of the Weir *et al.* (1984) phenological model optimized for the 210 genotypes of a wheat-association genetics panel when two (*V*_*sat*_ and *P*_*base*_; a, b) or three (*V*_*sat*_, *P*_*base*_, and *TT*_*emh*_; c, d, and e) parameters were optimized. This figure is available in colour at *JXB* online.

In the *2p* analysis, *V*_*sat*_ varied from 6 to 127 days and *P*_*base*_ from 0 to 9h among genotypes. Based on Shapiro-Wilk tests, distributions of *V*_*sat*_ and *P*_*base*_ could not be considered as Gaussian (*P*-values <0.001 and <0.01, respectively). Both parameters showed multimodal distributions suggesting the presence of major genes with large effects segregating in this material (Fig. 2A and 2B). A significant but small positive correlation coefficient (*r*) was found between *V*_*sat*_ and *P*_*base*_ (*r* = 0.32, *P* < 0.001, data not shown).

In the *3p* analysis, *V*_*sat*_ varied from 7 to 130 days among genotypes; *P*_*base*_, from 0.1 to 8.8h; and *TT*_*emhe*_, from 400 to 740 modified degree days. The distributions of optimized parameters *P*_*base*_ and *TT*_*emhe*_ were skewed towards extreme values (7h and 400°C days, respectively; Fig. 2D and 2E). Moreover, the correlation between these two parameters was highly negative (*r* = –0.70, *P* < 0.001, data not shown), indicating possible compensations between these parameters and making it difficult to identify parameter vectors that independently reflect genotype photoperiod sensitivity and earliness *per se*.

### Prediction of heading date using the ecophysiological model with optimized parameters

Depending on the environment, the difference between the latest and the earliest heading date varied from 24 to 66 days, thus reflecting the large genotypic variability for heading date in this germplasm (Table 1). In autumn-sown experiments, this difference varied from 24 to 36 days while it was higher in spring-sown experiments, ranging from 60 to 66 days (Table 1).

**Table 1.** RMSEP and percentages of variance explained (R^2^) of the relationships between observed and predicted heading dates simulated with a modified version of the Weir *et al.* (1984) phenological model for the calibration and validation data sets grown in contrasting location × sowing date combinations using optimized (calibration data set only) or QTL-based parameters (calibration and validation data sets)^a^

Data set	Location	Sowing date	n	Mean (min.; max.)	Optimized parameters				QTL-based parameters			
					*2p*		*3p*		*2p*		*3p*	
					RMSEP	R^2^	RMSEP	R^2^	RMSEP	R^2^	RMSEP	R^2^
Calibration	Clermont- Ferrand	27/10/2004	206	139 (126; 154)	2.3 *(5.2*)	0.93 (*0.87*)	2.0	0.94	3.6	0.68	5.0	0.34
		08/11/2005	62	143 (133; 157)	3.7 (*2.4*)	0.93 (*0.89*)	3.0	0.94	4.5	0.68	5.3	0.31
		**23/02/2006**	**75**	**149 (140; 165)**	**5.5** (***8.1***)	**0.73** (***0.56***)	**3.5**	**0.84**	**8.2**	**0.41**	**8.7**	**0.27**
		20/11/2008	210	143 (130; 159)	2.4 (*3.6*)	0.92 (*0.84*)	1.9	0.94	4.3	0.62	5.5	0.32
	Le Moulon	20/10/2003	171	146 (125; 161)	2.0 (*10.0*)	0.97 (*0.95*)	1.4	0.98	4.2	0.76	6.3	0.42
		**05/04/2004**	**114**	**181 (160; 226)**	**5.4** (***7.9***)	**0.95** (***0.91***)	**5.0**	**0.95**	**17.6**	**0.63**	**14.6**	**0.68**
		20/10/2004	164	143 (121; 156)	1.9 (*8.1*)	0.96 (*0.90*)	1.5	0.97	4.1	0.73	5.9	0.41
		**04/04/2005**	**122**	**178 (160; 223)**	**4.0** (***6.2***)	**0.94** (***0.93***)	**4.1**	**0.94**	**13.4**	**0.56**	**10.6**	**0.62**
		20/10/2005	165	146 (132; 162)	1.5 (*8.6*)	0.96 (***0.91***)	1.6	0.96	4.3	0.69	6.5	0.34
		**07/04/2006**	**131**	**179 (160; 220)**	**2.6** (***4.8***)	**0.97** (***0.90***)	**2.5**	**0.97**	**15.0**	**0.54**	**15.4**	**0.48**
Validation	Le Moulon	26/10/2006	88	127 (100; 141)	-	-	-	-	5.6	0.59	6.6	0.38
		23/10/2007	88	142 (115; 157)	-	-	-	-	5.0	0.61	7.0	0.37
	Joze	29/10/2006	88	133 (112; 146)	-	-	-	-	5.7	0.57	7.7	0.31
		25/10/2007	88	147 (134; 160)	-	-	-	-	8.6	0.48	10.9	0.30
	Estrées- Mons	17/10/2006	88	135 (104; 151)	-	-	-	-	5.6	0.63	8.0	0.41
		22/10/2007	88	148 [129; 160)	-	-	-	-	6.7	0.58	9.0	0.31

^a^ Unusual spring sowings are highlighted in bold. Results obtained with optimized parameters on the calibration data set with the original model from Weir *et al.* (1984) are shown in parentheses. Results obtained after optimization of two (*2p*) or three (*3p*) parameters are shown. The number of wheat genotypes (n), the mean and the range of variation of heading dates (in days) are indicated. Genotypes and location × sowing date combinations of the validation data set were not used to optimize parameters or calibrate the QTL-based model and are therefore totally independent.

**Fig. 3.**
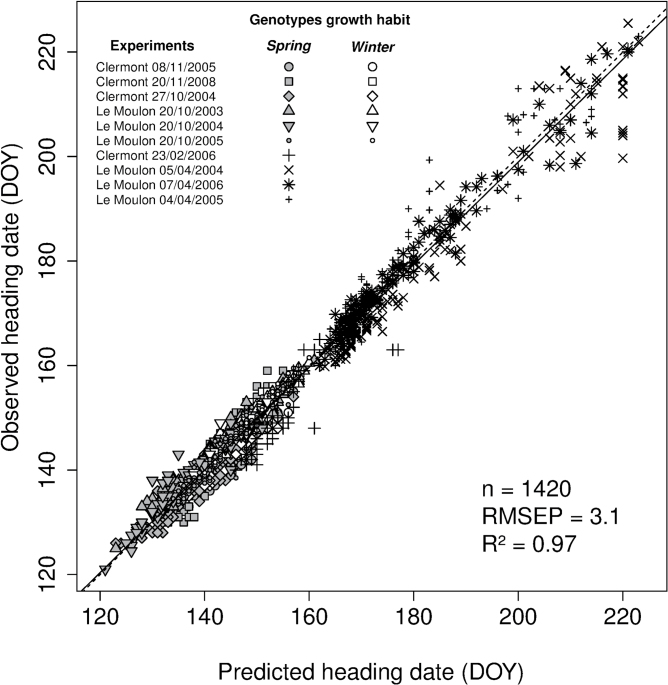
Relationship between observed and predicted heading dates obtained with two optimized parameters (*2p* strategy) of a modified version of the Weir *et al.* (1984) phenological model for a wheat calibration data set grown in 10 location × sowing date combinations. Autumn- and spring-sown experiments are shown with closed symbols and stars, respectively. Winter genotypes headed only in autumn-sown experiments while spring genotypes headed in autumn- and spring-sown experiments. Symbols for autumn-sown experiments are filled in white and grey for winter and spring genotypes, respectively. Linear regression (solid) and bisecting (dashed) lines are shown. The number of data points (n), the percentage of variance explained (R^2^) and RMSEP are indicated.

Across the 10 location × sowing date combinations, predicted heading dates obtained using the modified Weir *et al.* (1984) model with *V*_*sat*_ and *P*_*base*_ optimized parameters (*2p* strategy), explained 97% of the variation with an RMSEP of 3.1 days (Fig. 3). Separate analyses for the spring and winter genotypes showed RMSEP of 3.2 and 2.1, and R^2^ of 0.98 and 0.89, for these two groups of genotypes, respectively (data not shown). Considering each location × sowing date combination separately, RMSEP and R^2^ ranged from 1.5 to 5.5 days and 0.73 to 0.97, respectively (Table 1), thus showing that the model reproduced genotypic variability for heading date in the set of environments used for model calibration.

The modified *2p* strategy described above was also compared to a *2p* strategy using the original Weir *et al.* (1984) model, i.e. where thermal time was calculated using a three-hourly time step. The results with the original model showed an RMSEP ranging from 2.4 to 10 days with percentage of variance explained from 56 to 95% depending on the experiment (Table 1, numbers in parentheses), i.e. the modified model using thermal time based on daily mean temperature performed better than the original model, at least on this data set.

The results obtained with the *3p* strategy applied to the modified Weir model (optimization of *V*_*sat*_, *P*_*base*_, and *TT*_*emhe*_) were slightly better than those for the *2p* strategy modified Weir model, showing an RMSEP of 2.6 days and an R^2^ of 0.98 across all the 10 experiments of the calibration data set (data not shown). For the *3p* model, a separate analysis for the different experiments showed RMSEP ranging from 1.4 to 5.0 days and R^2^ from 0.84 to 0.98 depending on the experiment (Table 1).

### Association genetics analysis

For the *2p* strategy using the modified Weir *et al.* (1984) model, the genetic analysis identified 43 and 66 markers associated with *V*_*sat*_ and *P*_*base*_ (*P* < 0.05), respectively. The percentage of variance explained by each associated marker varied from 4 to 21% and 3 to 17% for *V*_*sat*_ and *P*_*base*_, respectively. Most of the markers (90%) associated with either *V*_*sat*_ or *P*_*base*_ explained less than 10% of the variation. Only markers that were co-located at known major gene loci (*Ppd-D1*, *Vrn-A1* and *Vrn-B1*) explained greater than 10% of parameter variations. No marker was associated with both *V*_*sat*_ and *P*_*base*_. All genomic regions associated to variations of *V*_*sat*_ and *P*_*base*_ had been previously detected as associated to variations of heading date in this panel (Bonnin *et al.*, 2008; Rousset *et al.*, 2011; Le Gouis *et al.*, 2012).

The results for the *3p* strategy identified 37, 2, and 3 markers associated with *V*_*sat*_, *P*_*base*_, and *TT*_*emhe*_ (*P* < 0.05), respectively. While the number of markers associated with *V*_*sat*_ appeared consistent between the *2p* (40 markers) and *3p* (37 markers) strategies, the number of genetic markers associated with *P*_*base*_ dropped between the *2p* (66 markers) and *3p* (two markers) strategies. The marker for the photoperiod-sensitivity gene *Ppd-D1*, which was previously associated with *P*_*base*_ in the *2p* strategy, was associated to *TT*_*emhe*_ in the *3p* strategy.

### QTL-based prediction of ecophysiological model parameters

**Fig. 4.**
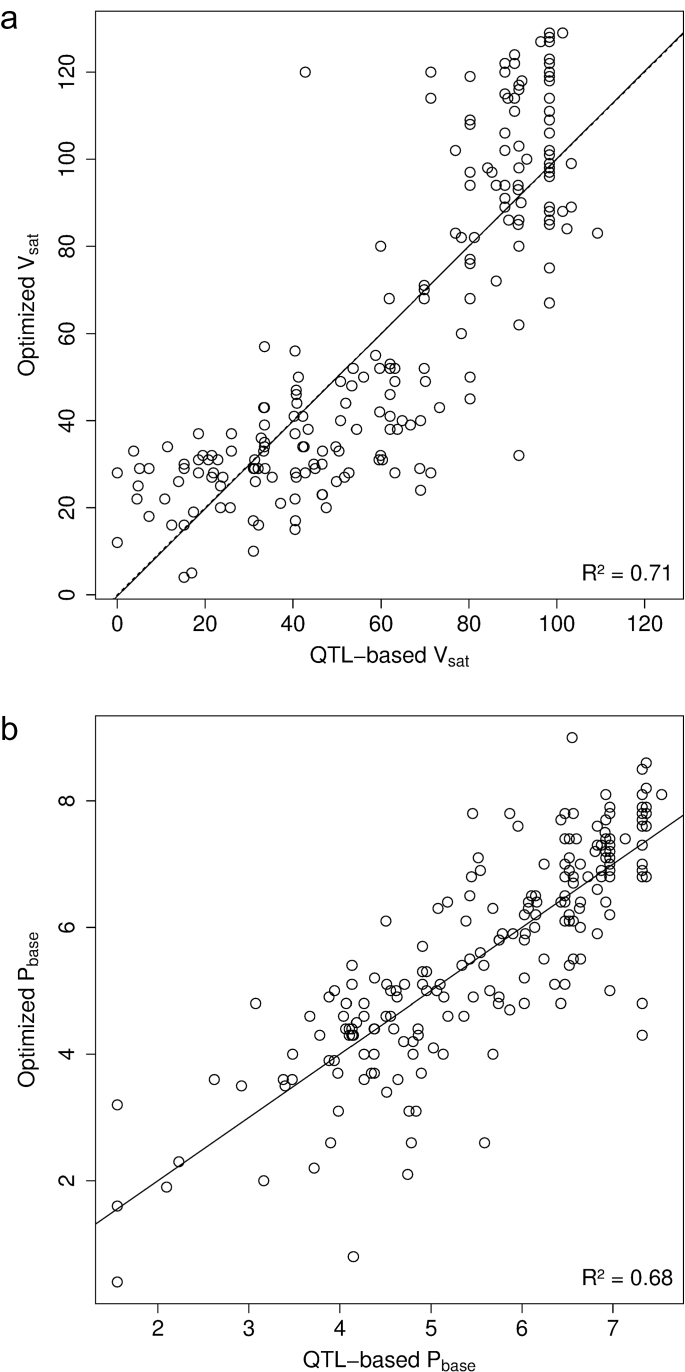
Relationships between optimized and QTL-based predicted *V*_*sat*_ (a) and *P*_*base*_ (b) parameters of a modified version of the Weir *et al.* (1984) phenological model for the 210 genotypes of the calibration data set. Solid lines are regression lines and R^2^ is the percentage of variance explained.

After filtering markers obtained with the *2p* strategy for collinearity, 28 and 36 markers were available to model *V*_*sat*_ and *P*_*base*_, respectively. The statistical model predicting *V*_*sat*_ comprised 11 markers located on chromosomes 2D, 4A, 4B, 5A, 5B, and 7A (Table 2) and explained 71% of the genotypic variation for *V*_*sat*_ (Fig. 4A). The most prominent markers in this model were those associated with *Vrn-A1* (markers Vrn.A1ex7 and Vrn.A1pr) and *Vrn-B1* genes (markers vern.5B.Sins.8761 and Vrn.B1int1) and one marker located on chromosome 2D (FdGogat.2D.Y.545). Markers Vrn.A1ex7, vern.5B.Sins.8761, Vrn.A1pr, and FdGogat.2D.Y.545 explained 39.5, 16.1, 4.6, and 5.3% of the variation of *V*_*sat*_ (Table 2). Two markers for each of the *Vrn-A1* and *Vrn-B1* genes were selected in the model, suggesting that there may be different polymorphic regions in these genes responsible for variations of vernalization requirement. Allelic effects (in absolute value) on *V*_*sat*_ ranged from 2.1 to 36.6 days (Table 2). Coefficients of the model for the alleles of the Vrn.A1ex7 marker agreed with the expectation that spring allele ‘22’ decreased the vernalization requirement (Table 2) (Rousset *et al.*, 2011). The statistical model predicting *P*_*base*_ comprised 12 markers located on chromosomes 1A, 2B, 2D, 3B, 5A, 5B, 6A, 6B, 7A, and 7B (Table 2) and explained 68% of the genotypic variation for *P*_*base*_ (Fig. 4B). The main explanatory markers were for the *Ppd-D1* gene (PpdD1.PromDel), an SNP (cfn5828), and an SSR (gpw1107), which explained 27.4, 15.3, and 12.2% of the variation of *P*_*base*_, respectively (Table 2). Allelic effects (in absolute value) on *P*_*base*_ ranged from 0.1 to 1.5h (Table 2). Coefficients of the model for the alleles of the PpdD1.PromDel marker agreed with the expectation that insensitive allele ‘2’ decreased the value of *P*_*base*_.

**Table 2.** Chromosome locations, allelic effects, standard errors (SE), *P*-values, and individual percentage of variance explained (*R* ^*2*^) of the markers used to predict the *V*_*sat*_ and *P*_*base*_ parameters of a modified version of the Weir *et al.* (1984) phenological model^a^

Parameter	Marker (reference allele)	Chromosome location	Allele	Allelic effects	SE	*P*-value	*R* ^*2*^ (%)
*V*_*sat*_	Intercept	-	-	53.9	9.2	<0.001	-
	Vrn.A1ex7 (11)	5A	12	–24.7	10	<0.05	39.5
			22	–28.3	4	<0.001	
	vern.5B.Sins.8761 (del)	5B	ins	13.3	5.7	<0.05	16.1
	Vrn.A1pr (11)	5A	22	7.0	5.3	0.19	4.6
			25	–29.9	15.1	<0.05	
			33	17.9	8.6	<0.05	
			44	36.6	11.1	<0.01	
			55	–1.7	6.5	0.80	
	FdGogat.2D.Y.545 (C)	2D	T	–9.4	3.9	<0.05	5.3
	Vrn.B1int1 (11)	5B	22	–13.1	5.8	<0.05	0.6
	cfn5274 (T)	2D	G	–6.6	4.0	0.10	1.4
	cfn5187 (T)	4A	C	2.1	4.3	0.62	1.1
	cfn5114 (T)	4A	C	8.0	4.2	0.06	0.6
	cfn5680 (A)	7A	G	7.0	3.5	<0.05	0.6
	wPt-7062 (0)	4B	1	10.0	4.2	<0.05	0.7
	cfn5405 (T)	na	C	–6.7	3.3	<0.05	0.6
*P*_*base*_	Intercept	-	-	4.85	0.4	<0.001	-
	PpdD1.PromDel (1)	2D	2	–1.5	0.2	<0.001	27.4
	cfn5828 (A)	2D	G	–0.5	0.2	<0.01	15.3
	gpw1107 (149)	3B	153	0.4	0.3	0.23	12.2
			169	0.5	0.3	0.15	
			171	0.7	0.4	0.12	
			173	0.1	0.3	0.83	
			175	–0.4	0.5	0.41	
			177	–0.1	1.1	0.94	
			179	–0.5	1.1	0.66	
	wPt-7063 (0)	6A	1	0.3	0.2	0.13	2.4
	cfn5539 (A)	2B	G	0.4	0.2	0.07	3.5
	cfn4818 (T)	7A	G	0.6	0.3	0.08	1.5
	cfn4764 (A)	5A	C	–0.5	0.2	<0.05	1
	cfn5634 (T)	6B	C	–0.4	0.2	<0.05	1
	cfn5055 (T)	5B	C	–0.4	0.2	0.05	0.9
	wPt-5346 (0)	5B	1	0.3	0.2	0.06	0.9
	gluA1.1.Y.1667 (C)	1A	T	0.4	0.2	<0.05	1.1
	wPt-7108 (0)	7B	1	–0.4	0.2	<0.01	1.2

^a^ Markers were identified by association genetics and chosen to be in low-linkage disequilibrium. Models were obtained using multiple linear regressions with iterative backward elimination of markers with *P*-values < 0.05. For each marker, the allele used as a reference to calculate the coefficient is indicated in brackets.na, not available.

Models obtained using only the known associated major genes for *V*_*sat*_ (*Vrn-A1*, *Vrn-B1*, and *LUMINIDEPENDENS*) and *P*_*base*_ (*Ppd-D1* and *Vrn-A3*) were calculated. Clearly, it appeared that prediction of *V*_*sat*_ was already reasonable when using only *Vrn-A1*, *Vrn-B1*, and *LUMINIDEPENDENS* as predictors (R^2^ = 0.61, data not shown) although the use of additional loci allowed reaching R^2^ = 0.71 (Fig. 4A). However, prediction of *P*_*base*_ using *Ppd-D1* and *Vrn-A3* was poor (R^2^ = 0.36, data not shown) and benefited from the inclusion of additional genetic markers (Fig. 4B).

For the *3p* strategy, 16 markers were identified for *V*_*sat*_, explaining 73% of the variation (data not shown). The model and QTL-based predictions of *V*_*sat*_ were very similar for the *2p* and *3p* strategies. However, the model for *P*_*base*_ comprised only one marker (wPt-1664, not associated to *P*_*base*_ in the *2p* strategy) and explained only 5% of *P*_*base*_ (data not shown). In the same way, model *TT*_*emhe*_ comprised only three markers (wPt-1664, *Ppd-D1*, and wPt-7108 associated to *P*_*base*_ in the *2p* strategy) and explained 25% of the variation for this parameter (data not shown).

### Predictions of heading date and model validation

**Fig. 5.**
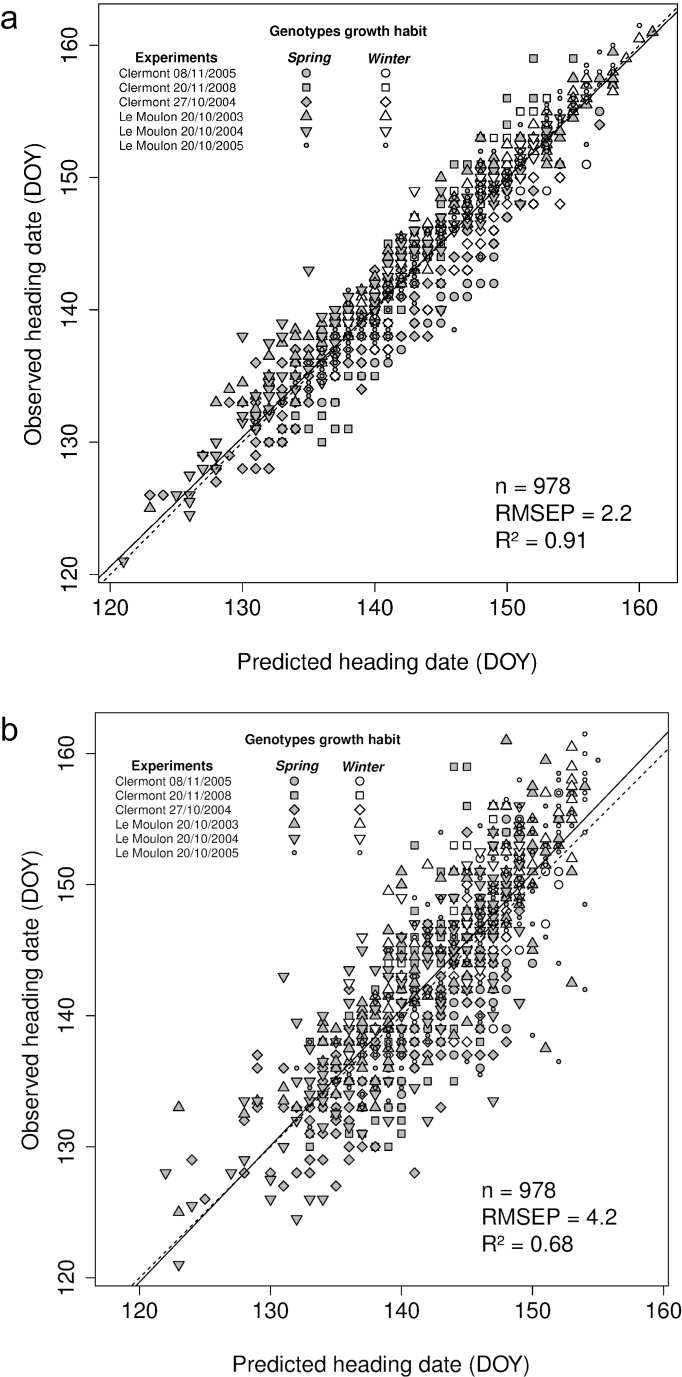
Relationships between observed heading dates and heading dates predicted with two optimized (a) or two QTL-based (b) parameters (*2p* strategy) for the calibration data set across the autumn-sown experiments using a modified version of the Weir *et al.* (1984) phenological model. Symbols are filled in white and grey for winter and spring genotypes, respectively. Linear regression (solid) and bisecting (dashed) lines are shown. The number of data points (n), the percentage of variance explained (R^2^), and RMSEP are indicated.

Observed heading dates were compared to heading dates predicted by optimized or QTL-based parameters obtained with the *2p* strategy for the autumn-sown experiments of the calibration data set. Across autumn-sown experiments of the calibration data set, predictions with optimized parameters explained 91% of the variation with an RMSEP of 2.2 days (Fig. 5A) while predictions with QTL-based parameters explained 68 % of the variation with an RMSEP of 4.2 days (Fig. 5B). Relationships between observed and predicted heading dates with optimized parameters across the autumn sown experiments showed RMSEP of 2.3 and 2.1 days and R^2^ of 0.90 and 0.89 for the spring and winter genotypes, respectively. In comparison, when QTL-based parameters were used, spring and winter genotypes showed RMSEP of 4.1 and 4.3 days and R^2^ of 0.66 and 0.67, respectively. This separate analysis did not show any prediction bias between the spring and winter genotypes. When considering individual experiments, R^2^ ranged from 0.92 to 0.97 (median equal to 0.94) and RMSEP from 1.5 to 3.7 days (median equal to 2.2) when heading dates were predicted with optimized parameters (Table 1) while R^2^ ranged from 0.62 to 0.76 (median equal to 0.68) and RMSEP from 3.6 to 4.5 days (median equal to 4.3) when heading dates were predicted with QTL-based parameters (Table 1). Compared to results obtained using optimized parameters, RMSEP was increased by 0.8 to 2.8 days (median increase of 2.0 days) and R^2^ decreased by 0.21 to 0.30 (median decrease of 0.25) when heading date was predicted using QTL-based parameters.

**Fig. 6.**
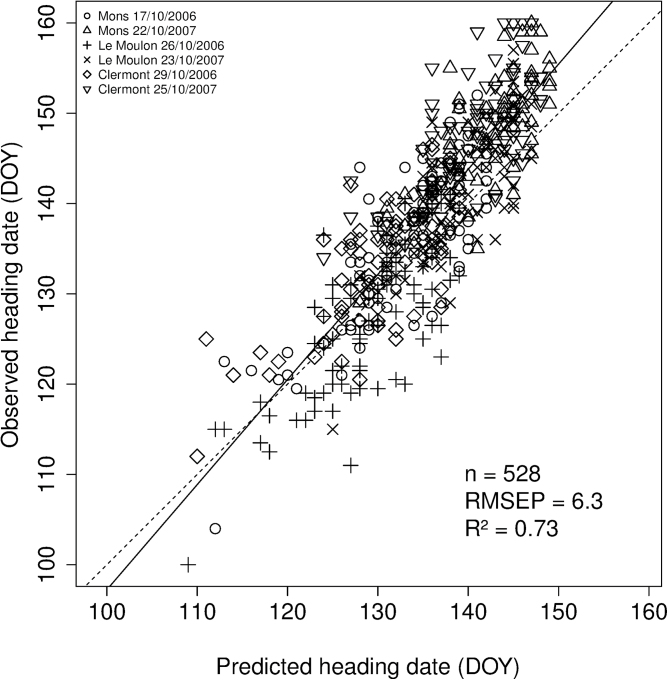
Relationships between observed heading dates and heading dates predicted with two QTL-based parameters (*2p* strategy) using a modified version of the Weir *et al.* (1984) phenological model for the validation data set comprising 88 independent wheat genotypes grown in six independent location × sowing date combinations. Linear regression (solid) and bisecting (dashed) lines are shown. The number of datapoints (n), the percentage of variance explained (R^2^), and the RMSEP are indicated.

The model was next tested on a set of 88 independent genotypes grown in six independent location × sowing date combinations. Differences between the earliest and the latest genotypes varied from 26 to 47 days in this validation data set (Table 1). This largely covered the range of variation observed in the calibration data set for autumn sowings which varied between 24 and 36 days (Table 1). Across all of the validation data set, heading dates predicted with QTL-based parameters explained 73% of the variation with an RMSEP of 6.3 days (Fig. 6). When looking at each location × sowing date combination of the validation data set separately (Table 1), RMSEP ranged from 5.0 to 8.6 (median equal to 5.6 days) and R^2^ from 0.48 to 0.63 (median equal to 0.58).

In comparison to the *2p* strategy, QTL-based prediction results obtained with the *3p* strategy showed reduced predictive ability for both the calibration and validation data sets. Separate analyses for each autumn-sown experiment of the calibration data set showed RMSEP ranging from 5 to 6.5 days and R^2^ ranging from 0.31 to 0.42 (Table 1). Considering experiments of the validation data set separately, RMSEP ranged from 6.6 to 10.9 days and R^2^ from 0.30 to 0.41 (Table 1).

## Discussion

Gene-based modelling assists in integrating knowledge from plant ecophysiology and genetics (Chapman *et al.*, 2002a; Chapman *et al.*, 2002b; White and Hoogenboom, 2003; White, 2006; Letort *et al.*, 2008; White, 2009). One outcome of this is to improve ecophysiological modelling and the prediction of cultivar performance. Another outcome is to assist in quantitatively assessing the effect of genes by explicitly accounting for genotype × environment interactions. In terms of targeting breeding, these outcomes can open the way to the identification of ideotypes for current and/or future climate environments (Chenu *et al.*, 2009). In this study, we proposed a QTL-based ecophysiological model to predict heading date in bread wheat. Two parameters (*V*_*sat*_ and *P*_*base*_) of an ecophysiological model were optimized for each genotype of an association genetics panel representative of the wheat germplasm (Balfourier *et al.*, 2007; Rousset *et al.*, 2011). Multiple linear models predicting *V*_*sat*_ and *P*_*base*_ using associated genetic markers were obtained by stepwise regression. Predictions of heading dates using QTL-based parameters were tested on a data set with independent genotypes and environments. The conditions required to identify parameter vectors which reflect genotypic differences and the ability to predict heading date using QTL-based parameters are discussed.

### Identifying parameter vectors reflecting genetic differences

The first condition for a successful gene-based modelling approach is to use an ecophysiological model with adequate predictive capabilities. In particular, the ability of the model to deal with complex genotype × environment interactions relevant to the studied process is a key feature. The ecophysiological model used in this study was a modified version of a model proposed by Weir *et al.* (1984), with a simplification of the calculation of accumulated daily temperature. The original formulation relies on the simulation of a cosinusoidal variation of temperature across the day from daily minimal and maximal temperature data. Temperature across the day is then integrated by averaging the contributions of the eight three-hour temperatures each day [equation 1 in Weir *et al.* (1984)]. In the present study, as in other major wheat crop models Sirius (Jamieson *et al.*, 1998) and APSIM (Keating *et al.*, 2003), we used daily mean temperature as an input and therefore ignored temperature variation across the day. The two formulations may give different results because of the non-linearity of the response curves used. However, the formulations used by Weir *et al.* (1984) and the one used in the present study are extremely empirical and do not reflect the current knowledge on the underlying physiological processes (Brown *et al.*, 2013). Hence, the use of the original or the modified model can only be justified by comparing their predictive power. Results obtained with the present model and the original Weir *et al.* (1984) model indicated that calculation of thermal time accumulation using daily mean temperature performed better than using the original formulation (Weir *et al.* 1984), at least on this data set (Table 1). We did not investigate further why our degraded version of the model performed better with our data set as this was not the primary objective of our study.

We also modified the original model by suppressing the intermediate phase from emergence to double-ridge and by assuming that thermal time accumulation from emergence to heading was affected by vernalization and photoperiod factors throughout the phase. This is an oversimplification as vernalization mainly affects the phase from emergence to double-ridge (Ritchie, 1991), although it may also have minor effects from double-ridge to heading (Griffiths *et al.*, 1985). These modifications were applied because no observations of double-ridge were available, and the model could not be parameterized for this growth stage. Again, while still quite empirical, the model showed good capability in predicting heading dates of an extremely diverse genetic panel with contrasting sowing dates (Table 1). Prediction errors were comparable to the ones reported by previous studies, which varied from 4 to 7 days (Weir *et al.*, 1984; Asseng *et al.*, 1998; White *et al.*, 2008; He *et al.*, 2011).

The second condition for a successful gene-modelling approach relies on an ability to unambiguously parameterize the model for a large number of genotypes. Most of the studies linking genetic markers and parameters of an ecophysiological model were carried out after measuring parameters on a mapping population (Reymond *et al.*, 2003; Nakagawa *et al.*, 2005; Quilot *et al.*, 2005; Yin *et al.*, 2005; Uptmoor *et al.*, 2011). Direct measurement of parameters allows unambiguous identification of parameter vectors but is feasible only in the case of mechanistic models for which parameters have a clear biological meaning. Only few studies have been carried out using optimized parameters (Messina *et al.*, 2006; White *et al.*, 2008), which is less demanding on phenotyping. However, parameter optimization is generally not straightforward and sometimes depends on subjective decisions (Mavromatis *et al.*, 2001) as different parameter combinations may equally fit the relationships between observations and model predictions. Although this is not an issue when the objective is only to predict heading dates, this becomes an obstacle when the objective is to link parameters to genes.

The dimension of parameter space, which depends on the number of parameters to be optimized and the range of variation of each parameter, contributes to both accurate and unambiguous parameterization. Indeed, in the *3p* strategy, compensations between *P*_*base*_ and *TT*_*emhe*_ led to multiple solutions and parameter vectors could not be unambiguously identified. This was not the case when only *V*_*sat*_ and *P*_*base*_ were optimized (*2p* strategy). Moreover, the restricted parameter space used in this study allowed optimizing parameters by calculating RMSEP for all the parameter combinations (brute-force optimization), an approach that allows a full exploration of parameter space. More-complex traits depending on a higher number of model parameters would probably need to use optimization algorithms, ideally independent of starting values and able to avoid local minima (He *et al.*, 2011).

The use of phenotypic data recorded under various experimental conditions for which model sensitivity varied greatly (Fig. 1) also contributed to unambiguous identification of parameter vectors. In this study, wheat genotypes were tested under contrasting sowing dates ranging from the usual autumn sowings corresponding to local agronomic practices under these latitudes to the most extreme spring sowings under which winter genotypes did not head due to incomplete vernalization. The use of srping-sown experiments, either to reveal the strong winter nature of some genotypes that did not head or more generally to quantify accurately genetic variation for vernalization requirements, assisted in accurately optimizing genotype parameters. Other experiments carried out in different latitudes or under controlled photoperiodic or vernalizing conditions could assist in optimizing genotype parameters if the simple ecophysiological model used in this study proved valid for these experiments where non-natural rapid changes in photoperiod and temperature regimes are created.

A third condition for a successful gene-based modelling approach is to develop a clear and robust genetic determinism to optimized parameter values. Reducing the number of parameters to calibrate the model may have led to a dilution of the genetic effects by increasing the number of genes involved in the determinism of each parameter. Among the markers associated with *V*_*sat*_ and *P*_*base*_, none were common to the two parameters, reflecting the fact that the model was able to separate the two individual physiological processes linked to vernalization and photoperiod. This was not the case when Le Gouis *et al.* (2012) estimated vernalization requirement and photoperiod sensitivity based on differences of heading dates under specific experiments designed to separate earliness components, but without using an ecophysiological model. In the gene network proposed by Trevaskis *et al.* (2007), vernalization and photoperiod perception are indeed independent: exposure to cold temperatures (vernalization) induces *VRN1* in the leaves, but *VRN1* expression at the apex remains low until *Ppd-D1* is induced by long days. It may also be hypothesized that as many genes will be involved in their determinism if the number of parameters is low, a larger proportion of parameter variation may be explained by interactions between markers rather than by marker additive effect. In our case, however, bi-locus marker × marker interactions were either non-significant or small (approximately 1% of *V*_*sat*_ variation explained for the most significant epistatic interaction between *Vrn-A1* and *Vrn-B1*; data not shown).

### QTL-based predictions of heading date

From a biological point of view, the values of *V*_*sat*_ and *P*_*base*_ may appear unrealistic, particularly where *V*_*sat*_ exceeds 80 days for some genotypes. Two possible explanations are the reduced number of parameters used to calibrate the model for different genotypes and the formulation of the model itself.

In the *2p* strategy, the absence of a parameter representing genetic variation for earliness *per se* implied that genetic variation for this earliness component would be included into *V*_*sat*_ and *P*_*base*_. The same approach with three parameters (*3p* strategy) appeared attractive since the three classical components of wheat earliness would have been represented by three different model parameters. However, comparison of the QTL-based predictions obtained with the *2p* and *3p* strategies clearly showed that the *2p* strategy performed better. The main objective of the present study was to maximize the predictive power of the QTL-based model using temperature and photoperiod environmental information, rather than to create a model formulation that would specifically coincide with actual knowledge of wheat earliness.

A final consideration is that the effect of the *V*_*sat*_ parameter may have been distorted due to the model itself. In the original model of Weir *et al.* (1984), the accumulated thermal time from emergence to double-ridge is modified by vernalization and photoperiod, but the following period from double-ridge to heading is only affected by photoperiod. As we could not calibrate this intermediate phase (due to absence of measurements), vernalization affects wheat development from emergence to heading in our model. However, despite these unrealistic values and its high empiricism, our QTL-based model was able to predict genotype variations for heading date across contrasting autumn sowings in different locations of France (Table 1, Fig. 6).

Association genetics did not reveal any novel loci compared to studies on heading date carried out with this plant material (Rousset *et al.*, 2011; Le Gouis *et al.*, 2012; Bordes *et al.*, 2013), i.e. in this case and contrary to other studies which investigated more complex traits (Reymond *et al.*, 2003; Quilot *et al.*, 2005), working on model parameters did not identify new chromosomal regions. However, contrary to studies carried out on heading date, this approach developed estimated effects that were independent of the environment and therefore more useful in prediction of performance in environments other than those originally tested. Indeed, contrary to classical QTL-analysis studies in which the effect of a QTL detected in different environments sometimes varies considerably, even for major genes like *Ppd-D1* (Bogard *et al.*, 2011), this approach led to a single value for *V*_*sat*_ and *P*_*base*_ taking into account all the environments. Given results obtained on a set of independent genotypes and environments (Table 1, Fig. 6), the heading dates of any allelic combination can be simulated with the QTL-based ecophysiological model with a median prediction error of 5.6 days, with the expectation that predicted heading dates would explain 60% of the variation for this trait.

However, as the multiple linear regression models for *V*_*sat*_ and *P*_*base*_ explained only 71 and 68% of the variation of these parameters, about one third of their genetic variation remains unexplained. The use of genetic markers in linkage disequilibrium as proxies instead of markers in the causal polymorphism may have reduced the ability to predict *V*_*sat*_ and *P*_*base*_ due to possible recombinations. Moreover, the presence of multiple alleles for *Ppd-B1* and *Ppd-D1* could be taken into account to improve our estimation of the genotype *P*_*base*_ value, i.e. one improvement would be to use diagnostic markers for these genes (Guo *et al*., 2010; Díaz *et al*., 2012; Shaw *et al*., 2012; Cane *et al*., 2013). In addition to errors coming from the model itself, which is quite simple, the remaining unexplained genetic variation may also be attributed to the fact that small-effect loci could not be detected by association genetics due to insufficient size of the panel or insufficient coverage of the wheat genome. Contrary to maize, where no major genes but approximately 50 small-effect additive QTLs with large polymorphism regulate flowering (Buckler *et al.*, 2009), the genetic architecture of wheat flowering appears to comprise relatively few major genes which have, as in *Arabidopsis* (Salomé *et al.*, 2011), a large effect. However, our results show that small-effect loci are likely to be important in terms of prediction. This was shown when using only major genes to model *V*_*sat*_ (*Vrn-A1*, *Vrn-B1*, and *LUMINIDEPENDENS*, R^2^ = 0.61) and in particular for *P*_*base*_ (*Ppd-D1*, *Vrn-A3*, R^2^ = 0.36). Identification of additional minor loci would probably require a larger association panel and/or higher-resolution coverage of the genome. In a study of the same germplasm, Le Gouis *et al.* (2012) estimated genome coverage of about 60% with 760 markers. Although the number of markers used in our study was much higher (1603) and genome coverage was therefore expected to be >60%, it is not possible to guarantee full genome coverage as the total number of markers may have increased without a proportional increase in information. High-throughput DNA chips for wheat containing thousands of SNPs derived from ISBP would probably assist in resolving this issue (Paux *et al.*, 2010).

## Conclusions

Prediction of the parameters *V*_*sat*_ and *P*_*base*_ using 11 and 12 genetic markers, respectively, allowed simulation of heading date with a median RMSEP of 5.6 days and accounted for approximately 60% of the genotypic variation for heading date in an independent data set of 88 genotypes grown in six location × sowing date combinations. Contrary to a study carried out on heading date, the use of an ecophysiological model allows an estimation of the allelic effects of genes independently of the considered environments as long as temperature data are available. This represents added value for breeders as simulations of heading date carried out for different allelic combinations in different environments can assist in determining the most suitable allelic combination for a given targeted population of environments (i.e. using a historical temperature record) and represents a first step in the *in silico* identification of ideotypes (Tardieu, 2003; Chenu *et al.*, 2009).

## Supplementary material

Supplementary data can be found at *JXB* online.

Supplementary Figure S1. Values of the *Vsat* (A) and *Pbase* (B) parameters of a modified version of the Weir *et al.* (1984) phenological model obtained for 210 genotypes after optimization using 10 different sets of experiments.

## Funding

This work was funded by the EU Framework 7 ADAPTAWHEAT project (FP7-KBBE-2011–5, led by Simon Griffiths, JIC, UK).

## Supplementary Material

Supplementary Data

## Abbreviations:

DOY: day of the year
RMSEP: root mean square error of prediction.
