# Supplementary material for: Predictions of heading date in bread wheat (Triticum aestivum L.) using QTL-based parameters of an ecophysiological model
Source: J Exp Bot. 2014 Aug 22;65(20):5849–65. doi: 10.1093/jxb/eru328 (PMC4203124; doi:10.1093/jxb/eru328)
Supplement: Supplementary Data [file supp_65_20_5849__index.html]

Predictions of heading date in bread wheat (Triticum aestivum L.) using QTL-based parameters of an ecophysiological model — Predictions of heading date in bread wheat (Triticum aestivum L.) using QTL-based parameters of an ecophysiological model — Predictions of heading date in bread wheat (Triticum aestivum L.) using QTL-based parameters of an ecophysiological model — Supplementary Data 

# Predictions of heading date in bread wheat (*Triticum aestivum* L.) using QTL-based parameters of an ecophysiological model

## Supplementary Data

Data files

**Files in this Data Supplement:**

- Supplementary Data - Supplementary Data
