# Supplementary material for: Predictions of heading date in bread wheat (Triticum aestivum L.) using QTL-based parameters of an ecophysiological model
Source: J Exp Bot. 2014 Aug 22;65(20):5849–65. doi: 10.1093/jxb/eru328 (PMC4203124; doi:10.1093/jxb/eru328)
Supplement: Supplementary Data [file supp_eru328_jexbot121640_file001.pdf]

A

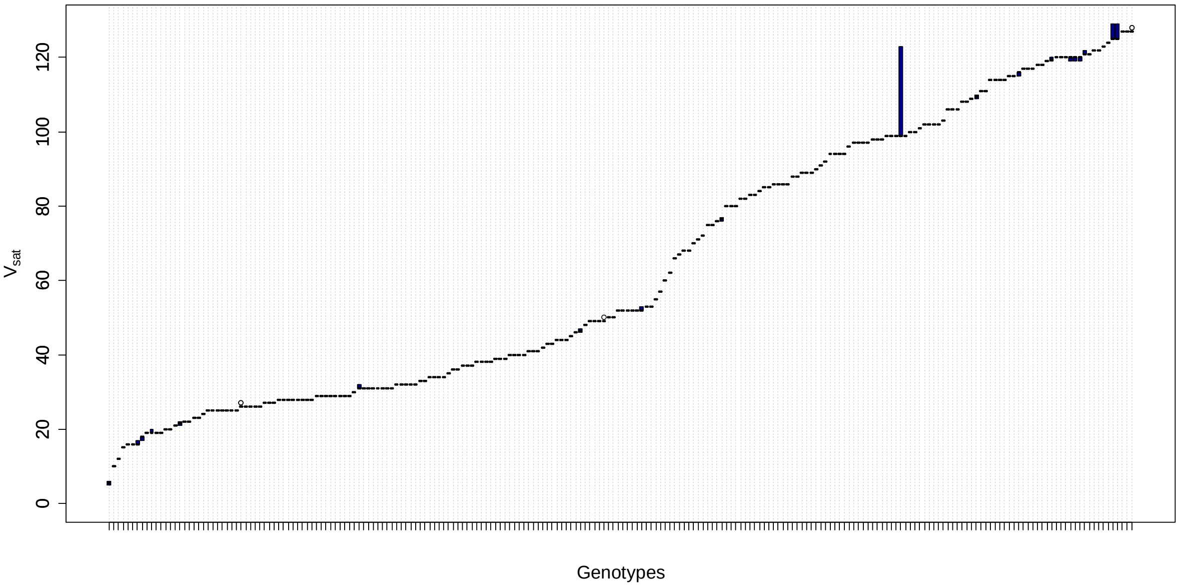

B

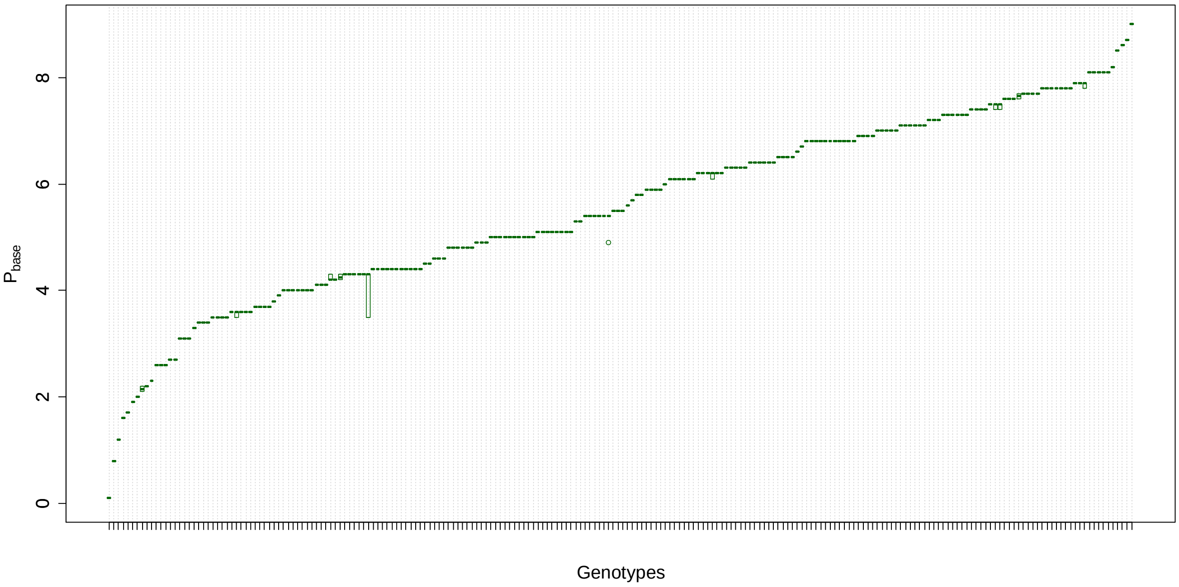

ESM Figure 1: Values of the V_{sat} (A) and P_{base} (B) parameters of a modified version of the Weir et al. (1984) phenological model obtained for 210 genotypes after optimization using 10 different sets of experiments. Each set of experiments was obtained for each genotype by sampling at random $n_{spring-1}$ of the spring sown experiments and $n_{autumn-1}$ of the autumn sown experiments (n_{spring} and n_{autumn} being the total number of available spring or autumn experiments for a given genotype). For each genotype, optimizations were carried out separately for each random set of experiments and results were combined to assess the robustness of the V_{sat} and P_{base} parameters.
